# Human Oncogenic Herpesvirus and Post-translational Modifications – Phosphorylation and SUMOylation

**DOI:** 10.3389/fmicb.2016.00962

**Published:** 2016-06-17

**Authors:** Pei-Ching Chang, Mel Campbell, Erle S. Robertson

**Affiliations:** ^1^Institute of Microbiology and Immunology, National Yang-Ming UniversityTaipei, China; ^2^UC Davis Cancer Center, University of California, Davis, DavisCA, USA; ^3^Department of Otorhinolaryngology and Tumor Virology Program, Abramson Cancer Center, Perelman School of Medicine at the University of Pennsylvania, PhiladelphiaPA, USA

**Keywords:** herpesvirus, post-translational modifications (PTMs), innate immunity, sumoylation, PML

## Abstract

Pathogens, especially viruses, evolve abilities to utilize cellular machineries to facilitate their survival and propagation. Post-translational modifications (PTMs), especially phosphorylation and SUMOylation, that reversibly modulate the function and interactions of target proteins are among the most important features in cell signaling pathways. PTM-dependent events also serve as one of the favorite targets for viruses. Among the seven unambiguous human oncogenic viruses, hepatitis B virus (HBV), hepatitis C virus (HCV), Epstein-Barr virus (EBV), Kaposi’s sarcoma-associated herpesvirus (KSHV), human papillomavirus (HPV), Human T lymphotrophic virus-1 (HTLV-1), and Merkel cell polyomavirus (MCPyV), two are herpesviruses. The life cycle of herpesviruses consists of latent and lytic phases and the rapid switch between these states includes global remodeling of the viral genome from heterochromatin-to-euchromatin. The balance between lytic replication and latency is essential for herpesvirus to maintain a persistent infection through a combination of viral propagation and evasion of the host immune response, which consequently may contribute to tumorigenesis. It is no surprise that the swift reversibility of PTMs, especially SUMOylation, a modification that epigenetically regulates chromatin structure, is a major hijack target of the host for oncogenic herpesviruses. In this brief review, we summarize the varied ways in which herpesviruses engage the host immune components through PTMs, focusing on phosphorylation and SUMOylation.

## Post-Translational Modifications (PTMs)

Post-translational modifications are reversible events that hold the key for the determination of protein function and increase the functional diversity of proteins. Phosphorylation, one of the most well-studied PTMs, regulates signal transduction through recruitment of different functional partners. Tyrosine phosphorylation recruits Src homology 2 (SH2) or phosphotyrosine binding (PTB) domain-containing proteins to propagate signaling events. Phospho-serine/threonine can be recognized by protein modules including 14-3-3 proteins, WW domains, WD40 repeats and the BRCT motif. Small ubiquitin-like modifier (SUMO) is another important PTM that was initially identified as an antagonist of ubiquitin (Ub). Ub is a small peptide that was the first identified protein in Ub-like (UBL) proteins family and labels protein through a conjugation system comprising E1 activation, E2 conjugation, and E3 ligation enzymes. Proteins carrying Lys-48 poly-Ub chains are targeted to the proteasome and degraded (Komander and Rape, 2012). Proteins carrying Lys-63 poly-Ub chains function as a scaffold to recruit proteins containing Ub-binding domains (UBDs; Chen and Sun, 2009). Similarly, SUMO-interacting motifs (SIMs) mediate the recognition of SUMOylation and enables SUMO modifications to serve as a platform to recruit additional proteins. Functioning as a scaffold for DNA-binding proteins, SUMOylation mediates epigenetic regulation of chromatin remodeling and transcription regulation. Thus, SUMO has been one of the most intensively studied PTMs in the context of chromatin-associated processes.

Many of the important immunomodulatory proteins and chromatin remodeling molecules are functionally dependent on and regulated by PTMs, primarily phosphorylation and SUMOylation, respectively. Many cellular antiviral defense systems recognize DNA viruses and limit their replication and spread. It is not surprising that oncogenic herpesvirus, including Epstein-Barr virus (EBV) and Kaposi’s sarcoma-associated herpesvirus (KSHV), encode proteins that control cellular PTM pathways to help their life cycle and evade host immune responses [reviewed in (Wimmer et al., 2012; Everett et al., 2013)]. The objective of this review is to focus on the current knowledge on oncogenic γ-herpesvirus and phospho- and SUMO modifications in immune regulation.

## PTM and Immune Function

Oncogenic herpesviruses include EBV and KSHV. EBV mainly infects B cells and causes lymphomas. KSHV infection is associated with Kaposi’s sarcoma (KS), primary effusion lymphomas (PEL), and multicentric Castlemen’s disease (Wen and Damania, 2010). Herpesvirus enters into host cells via two mechanisms: (i) direct membrane fusion: attachment and fusion of the viral envelope with the cell plasma membrane or (ii) endosome-dependent entry: receptor mediated binding and internalization through interactions between viral envelope glycoproteins with host cell surface molecules. Following capsid disassembly, the viral DNA is released (Chandran, 2010). In response to viral infection, the host innate immune system is activated to control the virus. Innate immunity is the first-line defense system of the host directed against virus through recognition of pathogen-associated molecular patterns (PAMPs) by pattern recognition receptors (PRRs). Exogenous viral DNA in endosomes is recognized by Toll-like receptors 9 (TLR9). Free exogenous viral DNA in the cytosol is recognized by either Z-DNA binding protein 1 (ZBP1), absent in melanoma 2 (AIM2), or cGAMP synthase (cGAS). Increasing evidence has shown that promyelocytic leukemia protein-nuclear body (PML-NB) may also mediate intrinsic immunity against viral DNA in the nucleus.

### Endosomal Toll-Like Receptors Mediated Immune Response

**FIGURE 1:**
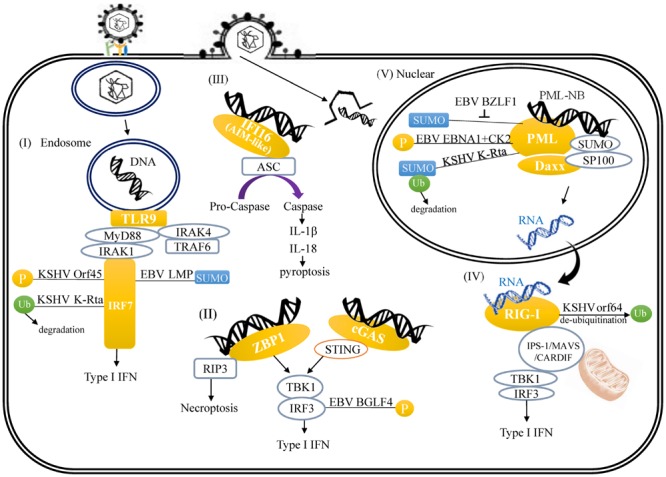
**A schematic illustrating herpesvirus DNA and RNA-induced innate immune response and the links between viral protein and host innate immune regulators. (I)** TLR9-mediated recognition of cytosolic viral double-stranded DNA (dsDNA) in the endosome. **(II)** ZBP-1 binds viral DNA in cytoplasm. **(III)** Activation of AIM2 inflammasome by viral DNA through binding of viral DNA to AIM-like receptor IFI16. **(IV)** RIG-I recognizes cytoplasmic viral RNA transcribed from herpesvirus DNA. **(V)** Modulation of PML-NB by herpesvirus-encoded proteins.

In response to DNA viruses, endosome-bound TLR9 induces a MyD88-dependent signaling with MyD88 forming complexes with IRAK-1, IRAK-4 and interferon regulatory factor-7 (IRF-7), which mediate the activation of type I interferon (IFN) pathway. Similar to other transcription factors (TFs), IRF-7 undergoes different PTMs, including phosphorylation, ubiquitylation, and SUMOylation. There are several examples of herpesvirus proteins targeting host IRFs. Open reading frame 45 (Orf45) of KSHV competitively inhibits IRF-7 phosphorylation, which is essential for IRF-7 transactivation activity (Zhu et al., 2002; Liang et al., 2012). KSHV replication and transcription activator (K-Rta) possesses Ub E3 ligase activity that mediates ubiquitylation and subsequently proteasomal degradation of IRF-7 (Zhu et al., 2002). miR-K12-11 encoded by KSHV inhibits IRF-3 phosphorylation, which is responsible for IRF-3 activation, through targeting IKK𝜀 (Liang et al., 2011). For EBV, latent membrane protein 1 (LMP1) induces IRF-7 SUMOylation, that inhibits its transactivation activity (Bentz et al., 2012) (**Figure 1-I**).

### DNA and RNA-Induced Immune Reaction

Cytosolic exogenous double-stranded DNA (dsDNA) is recognized by and triggers both Z-DNA binding protein 1/DNA-dependent activator of IFN-regulatory factors (ZBP1/DAI) and cGAMP synthase (cGAS) signaling that ultimately activates IRF-3-dependent type I IFN response. ZBP1, the first cytosolic dsDNA sensor, recruits TANK-binding kinase 1 (TBK1) and IFN regulatory factor-3 (IRF-3), with the consequent induction of type I IFN genes (Takaoka et al., 2007). cGAS, a member of the nucleotidyltransferase family, binds to foreign dsDNA in the cytoplasm and catalyzes cyclic GMP-AMP (cGAMP) synthesis. cGAMP directly interacts with STING and induces the STING-TBK1 signaling cascade that subsequently activates IRF-3 and IFN-β production (Sun et al., 2013). In the ZBP1 and cGAS pathway, EBV BGLF4 kinase phosphorylates IRF-3 and inhibits the active IRF3 recruitment to ISREs and thus suppresses the type I IFN response (Wang et al., 2009) (**Figure 1-II**). Other inhibitory mechanisms employed by EBV which affect innate immunity via phosphorylation includes latent membrane protein 1 (LMP-1) interaction with non-receptor tyrosine kinase 2 (Tyk2); an interaction that inhibits Tyk2 phosphorylation and consequently prevents activation of IFN-α signaling (Geiger and Martin, 2006). Cytosolic exogenous DNA also triggers AIM2 signaling that activates inflammasome-dependent pyroptosis. For AIM2 signaling, though emerging reports show that recognition of herpesvirus genomes by AIM-like receptor γ-interferon-inducible protein 16 (IFI16) can induce acetylation of IFI16 and IFI16-mediated inflammasome assembly (Ansari et al., 2015; Dutta et al., 2015) (**Figure 1-III**), there is currently no evidence to indicate that IFI16 acetylation is targeted by herpesviral proteins. In addition to viral DNA, DNA virus encoded RNA can also be recognized by RNA sensors such as RIG-I, which mediates the type I IFN pathway against viral infection. RIG-I-mediated signaling is controlled by ubiquitylation of RIG-I. Orf64 encoded by KSHV is a tegument protein with deubiquitinase (DUB) activity. Orf64 suppresses RIG-I-mediated IFN signaling by reducing the ubiquitylation of RIG-I (Inn et al., 2011) (**Figure 1-IV**). This regulation is also observed in non-oncogenic herpesvirus. For example, Orf63 encoded by α-herpesvirus varicella zoster virus (VZV), the causative agent of chickenpox, inhibits type I IFN signaling through proteasome-dependent IRF-9 degradation (Verweij et al., 2015). All these existing findings suggest that targeting PTMs of various host pathogen recognition receptors (PRR) or their down-stream effectors is a common and efficient mechanism by which herpesviruses suppress host innate immunity and facilitate intracellular host survival.

### Promyelocytic Leukemia-Nuclear Body (PML-NB)

Accumulating evidence indicates that PML-NB, also known as nuclear domain 10 (ND10) or PML oncogenic domains (PODs), target herpesviruses by inhibiting viral transcription and replication. PML-NB is a highly SUMO-modified nuclear compartment that is permanently composed of PML, hDaxx, and Sp100. Other factors can only be identified at PML-NB under certain unique conditions. Soon after viral DNA enters the nucleus, PML-NB rapidly associate with viral genomes in a SUMO-dependent manner. It is thus not surprising that herpesviruses have evolved mechanisms to target the SUMO machinery as a means to counteract the PML-NB-mediated antiviral response. Here, we focus on reviewing permanent PML-NB components but also include few important PML-NB variable proteins, such as ATRX that are targeted by oncogenic herpesviruses. Modification of PML by SUMO is a prerequisite for proper PML-NB formation. EBV BZLF1, also known as Zta, is SUMO modified and competitively reduces the SUMOylation of PML and consequently induces PML-NB disruption (Adamson and Kenney, 2001) (**Figure 1-V**). EBV EBNA1, one of the nuclear proteins of EBV that is expressed during both latent and lytic phases, interacts with protein kinase CK2 and consequently increases PML phosphorylation, and subsequent polyubiquitylation and degradation (Sivachandran et al., 2010) (**Figure 1-V**). KSHV immediate-early protein K-Rta has recently been identified as a SUMO-Targeting Ubiquitin Ligase (STUbL) that degraded SUMOylated PML and dispersed PML-NBs during the viral lytic cycle (Izumiya et al., 2013) (**Figure 1-V**). Emerging evidence suggests that, in addition to silencing viral gene transcription, PML functions as a co-activator of the type I IFN pathway (Scherer and Stamminger, 2016). SUMO modification of PML may negatively regulate PML’s ability to activate IFN signaling (Maarifi et al., 2015), however the study of viral antagonism of the IFN-stimulating function of PML is still in its infancy. It has been documented in human cytomegalovirus (HCMV), a β-herpesvirus, IE1 protein encoded by HCMV counteracts the type I IFN response by sequestration of interferon-stimulated gene factor 3 (ISGF3; Kim and Ahn, 2015; Scherer et al., 2015).

## Human Oncogenic Herpesviral Proteins and PTMs

In addition to regulating PML by SUMOylation, many herpesviral proteins are themselves SUMO modified, as mentioned earlier for EBV BZLF1. Both of the EBV immediate-early proteins BRLF1/Rta (Chang et al., 2004) and BZLF1/Zta (Hagemeier et al., 2010; Murata et al., 2010) can be SUMO modified and SUMOylation is involved in the regulation of their transactivation activity. EBV latent protein EBNA3C can also be SUMO modified and this SUMOylation is essential for EBNA3C coactivation activity with EBNA2 (Rosendorff et al., 2004). Differing slightly from EBV, among the two immediate-early proteins of KSHV, only K-bZIP, but not K-Rta, is efficiently modified by SUMO (Izumiya et al., 2005). Interestingly, KSHV K-Rta itself is a STUbL that mediated SUMO-dependent degradation of SUMOylated viral and cellular proteins (Izumiya et al., 2013). On the other hand, EBV BRLF1/Rta interacts with the cellular STUbL, RNF4, and is regulated by RNF4 (Yang et al., 2013). KSHV K-bZIP is not only SUMO modified, but also been recently identified as a viral SUMO E3 ligase that can SUMO modify many cellular proteins, including p53, IRF-1, and IRF-2 (Chang et al., 2010, 2013). SUMOylation and SUMO E3 ligase activity of K-bZIP are both involved in its transrepression activity (Izumiya et al., 2005; Yang et al., 2015). In addition to targeting IRFs by SUMOylation, KSHV encodes many viral IRFs (vIRF1–vIRF4) and vIRF3 was shown to be SUMO modified. SUMOylation of vIRF3 is required for its ability to disrupt PML-NB (Marcos-Villar et al., 2011). vIRF3 was also shown to interact with and inhibit the SUMO modification of tumor suppressors of pRb, p107, and p130 (Marcos-Villar et al., 2014). In addition, KSHV latent protein LANA is also SUMO modified and contains two SUMO interacting motifs (SIMs) within its N-terminus. SIMs of LANA are essential for LANA-mediated recruitment of SUMO-modified chromatin remodeling proteins including KAP-1 (Cai et al., 2013).

Chromatin remodeling protein KAP-1 can also be regulated by KSHV viral protein kinase (vPK). Phosphorylation of KAP-1 by vPK reduces KAP-1 SUMOylation and subsequently decreases the binding ability of KAP-1 on chromatin (Chang et al., 2009). Moreover, vPK also interacts with and phosphorylates KSHV K-bZIP. Phosphorylation of K-bZIP reduced the SUMOylation level and transrepression activity of K-bZIP (Izumiya et al., 2007). EBV BGL4 kinase mediated a serial phosphorylation, through TIP60 and ATM, to histone protein γ-H2AX and the chromatin remodeling protein KAP-1. This creates an open chromatin structure of viral episomes which facilitates viral lytic replication (Li et al., 2012). EBV BGL4 also mediates the phosphorylation of EBV BZLF1/Zta and consequently reduces the SUMOylation of BZLF1/Zta (Hagemeier et al., 2010).

## Conclusion and Future Prospects

Current knowledge of the mechanisms involved in viral infection and tumor progression is still largely unknown. Emerging evidence shows the importance of immune response in cancer. Here, we summarize how the host cells use PTMs, especially phosphorylation and SUMOylation, to regulate many important immunomodulatory proteins. To survive in the host, viruses express different proteins that target PTMs and consequently repress host immune defenses. The potential mechanisms used by oncogenic herpesvirus, including EBV and KSHV, are depicted. Studying the interplay between virus and host immune system will provide novel insight for development of future therapeutic intervention in cancer.

## Author Contributions

PCC and MC were involved in writing and editing of the review. ER was involved in paper construction and writing.

## Conflict of Interest Statement

The authors declare that the research was conducted in the absence of any commercial or financial relationships that could be construed as a potential conflict of interest.
